# Supplementary material for: Structure of formylpeptide receptor 2-Gi complex reveals insights into ligand recognition and signaling
Source: Nat Commun. 2020 Feb 14;11:885. doi: 10.1038/s41467-020-14728-9 (PMC7021761; doi:10.1038/s41467-020-14728-9)
Supplement: Supplementary file 1 — Supplementary Information [file 41467_2020_14728_MOESM1_ESM.pdf]

Supplementary Information

Structure of formylpeptide receptor 2-G_i complex reveals insights into ligand recognition and signaling

Youwen Zhuang, Heng Liu et al.

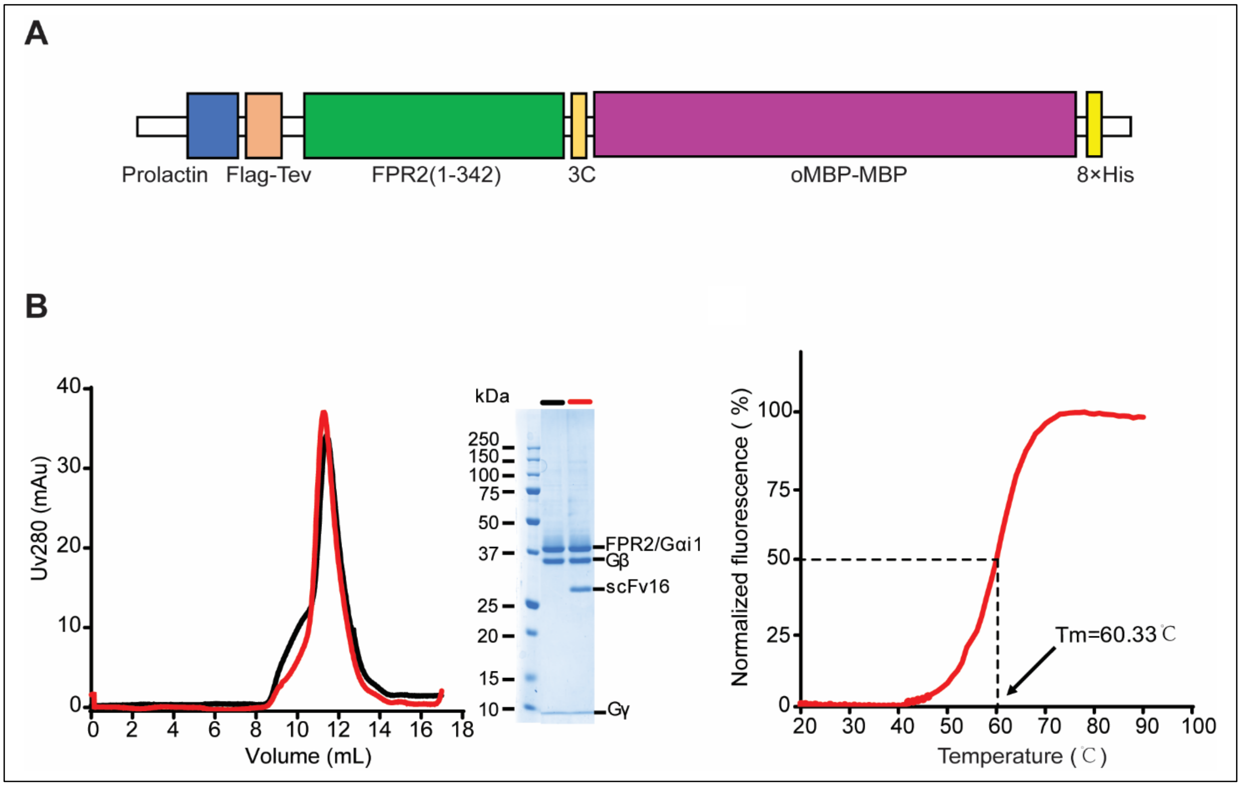

Supplementary Figure 1. Sample preparation. (A) Cartoon model of human FPR2 construct used in the study. It is cloned into pFastBac vector (Fisher). The final sample was treated with HIV 3C protease. Primer sequences for making this construct can be found in **Supplementary Table 3. (B)** Comparison of FPR2-G_{i1} complex with and without scFv16 by size exclusion chromatography (SEC) and SDS-PAGE. CPM assay of FPR2-G_{i1}-scFv16-WKYMVm complex was performed to determine half denaturing temperature Tm. Tm was determined by fitting the curve to Boltzmann sigmoidal equation (GraphPad).

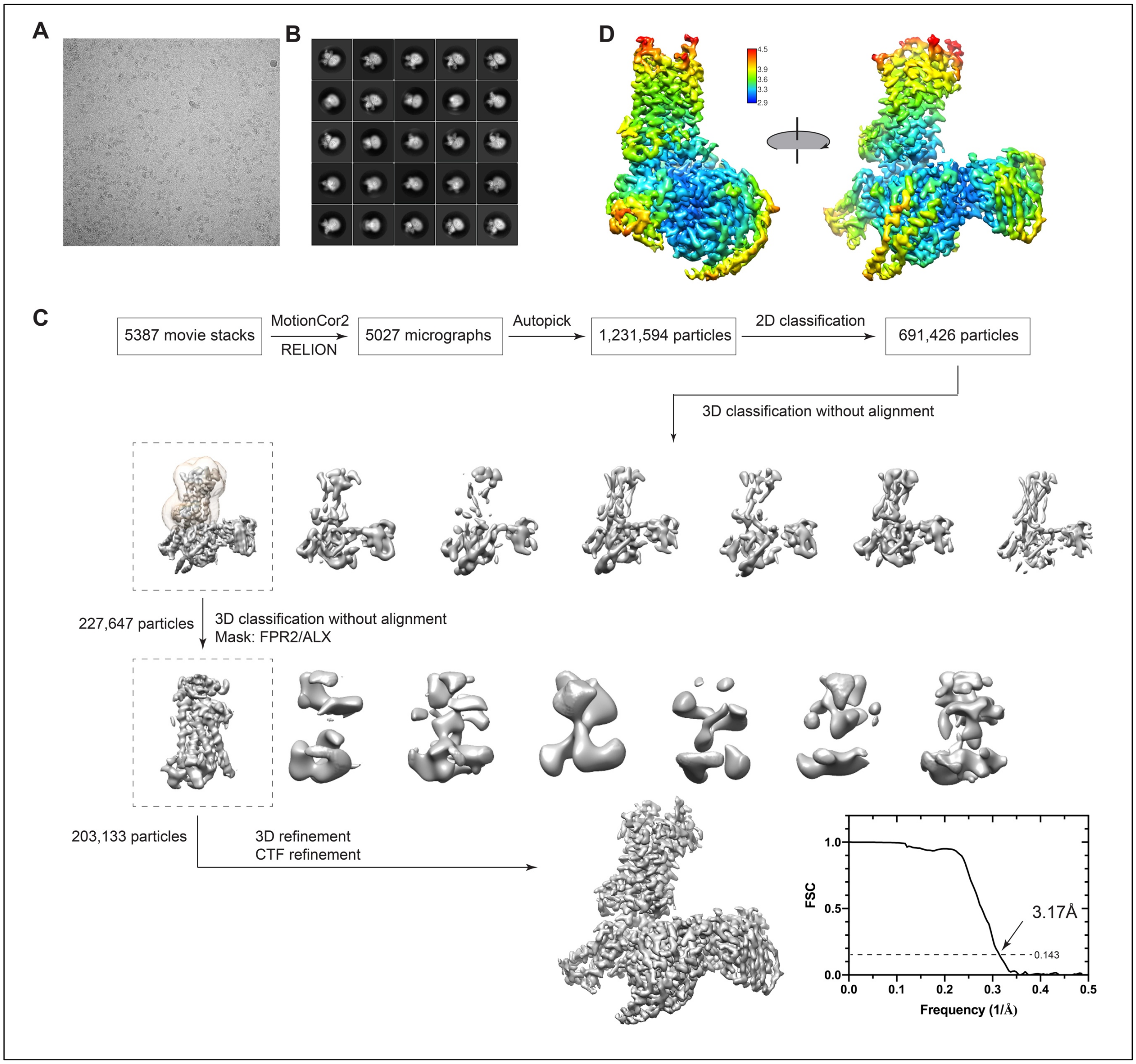

Supplementary Figure 2. Cryo-EM data collection, structure determination and cryo-EM maps. (A) Representative cryo-EM micrograph of FPR2-G_i-svFv16 complex. **(B)** 2D class averages of the complex in digitonin without reference. **(C)** Work flow of cryo-EM structure determination. The half-map Fourier Shell Correlation (FSC) plots generated by RELION is shown at the lower right corner. An overall resolution was set at 3.17-Å at 0.143 FSC. **(D)** Cryo-EM map colored according to local resolution (Å), which was calculated from half-maps by RELION.

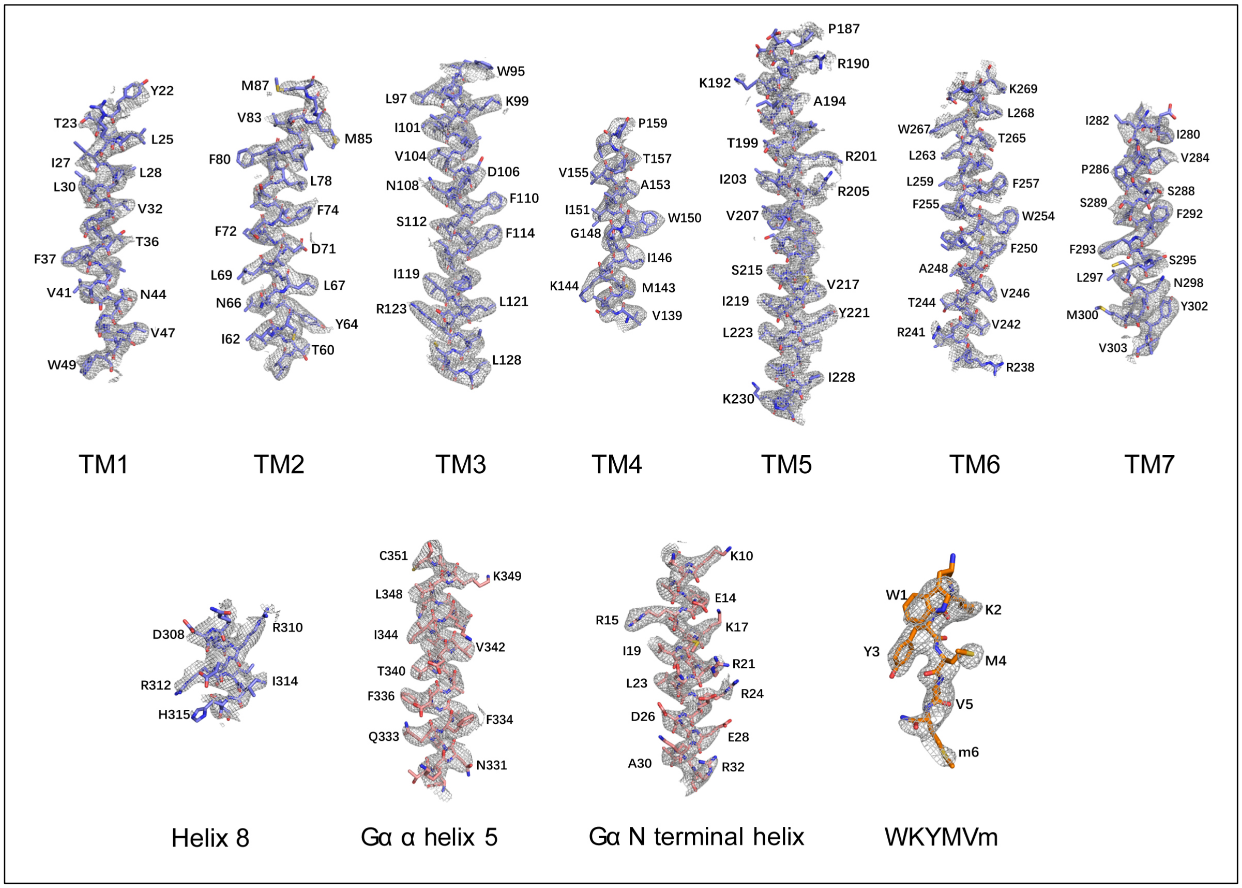

Supplementary Figure 3. Cryo-EM map of transmembrane regions TM1-TM7 and helix 8 of FPR2, αN and α5 helices of $G\alpha_i$ and the ligand WKYMVm.

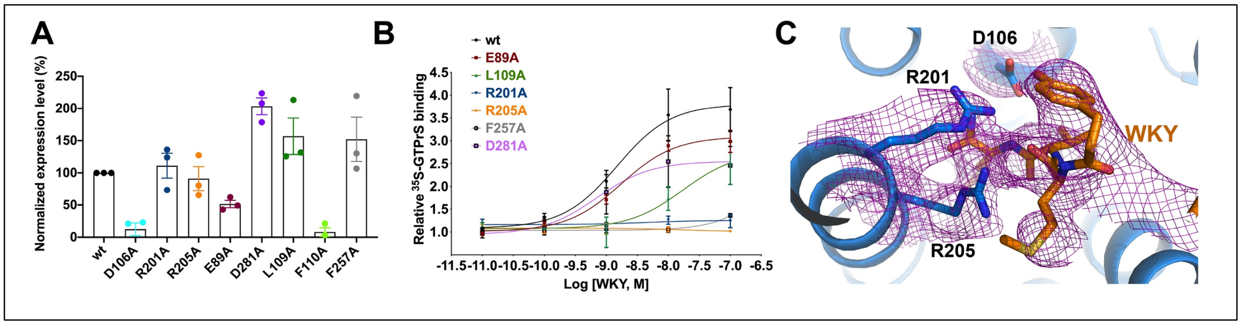

Supplementary Figure 4. Mutagenesis data to validate WKYMVm-binding mode. (A) Expression levels of FPR2 mutants in HEK293 cells determined by fluorescent antibody staining. **(B)** ^{35}S-GTPγS binding assays using membranes of HEK-293 cells overexpressing FPR2 mutants and purified G_i heterotrimer with increasing concentrations of WKYMVm (WKY). For wild type FPR2 (wt) and each mutant, the same amount of cell membrane and Gi protein were used in the assays. Mutants D106A and F110A with little cell surface expression were not tested in the binding assays. L109A, R201A, R205A, F257A mutations led to significantly compromised receptor activation induced by WKYMVm, even though their expression levels were similar to or higher than that of wtFPR2. All of these residues interact with WKYMVm. The reduction in cell surface expression of E89A mutant makes it difficult to interpret the associated reduction in receptor activation. **(A)** and **(B)** represent results from 3 independent assays and the data is shown as mean ± s.e.m. **(C)** Cryo-EM map of residues D106$^{3.33}$, R201$^{5.38}$ and R205$^{5.42}$ and part of WKYMVm. Source data are provided as a Source Data file.

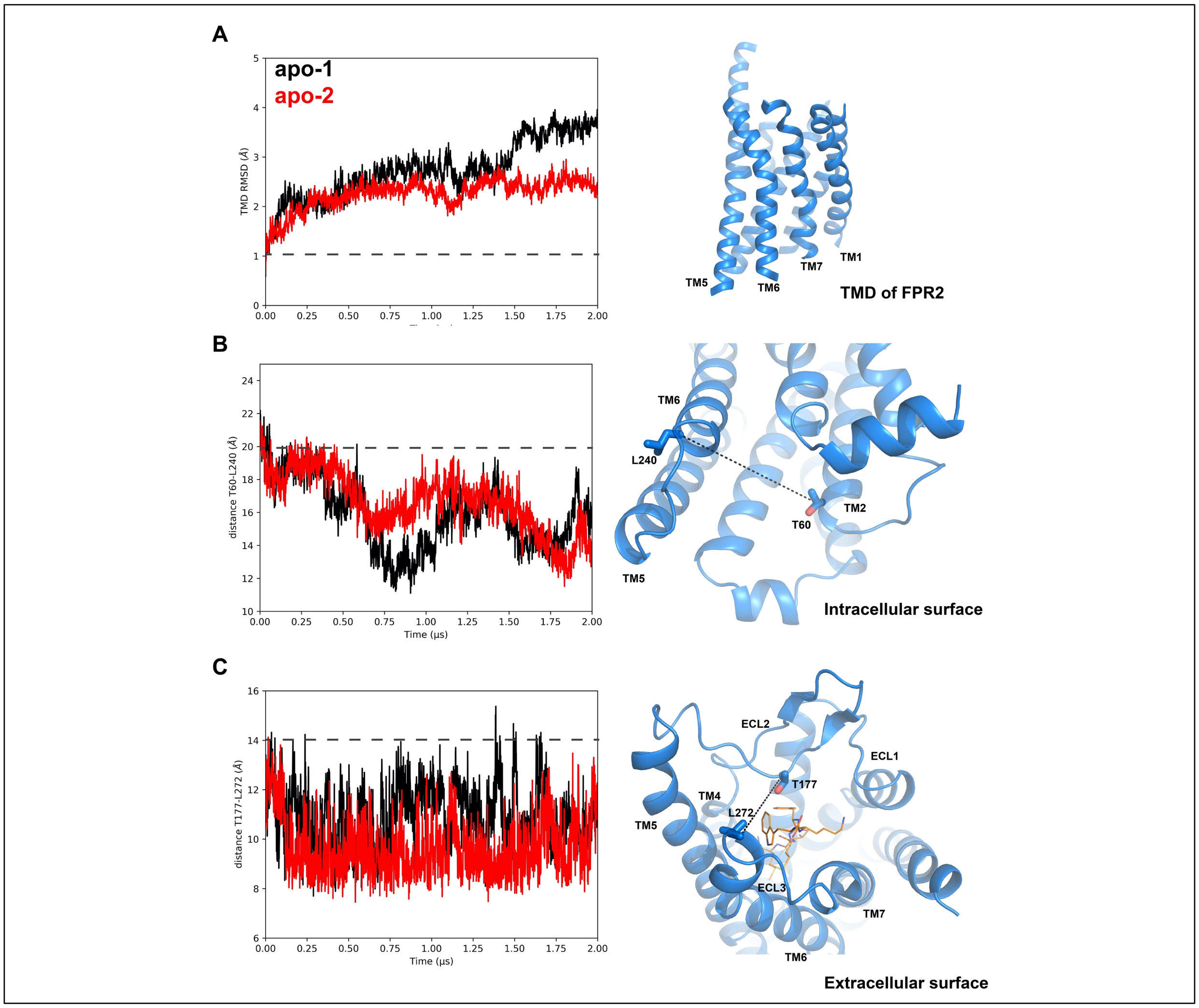

Supplementary Figure 5. Conformational changes of FPR2 in molecular dynamics (MD) simulations. Apo-1 (black traces) and apo-2 (red traces) represent two simulations of apo-FPR2 initiated from the cryo-EM structure with the ligand and G_i protein removed in a timescale of 2 μs. Dashed lines indicate the starting values in the cryo-EM structure. **(A)** RMSD of the transmembrane domain (TMD) of FPR2 in simulations compared to that in the cryo-EM structure. **(B)** Distance between the end of TM6 (L240) and TM2 (T60) in simulations. **(C)** Distance between ECL3 (represented by L272) and ECL2 (represented by T177) in simulations. In both simulations, the end of TM6 moved towards TM2 and ECL3 and ECL2 got closer to each other. ECL2 and ECL3 also showed large conformational fluctuations in simulations.

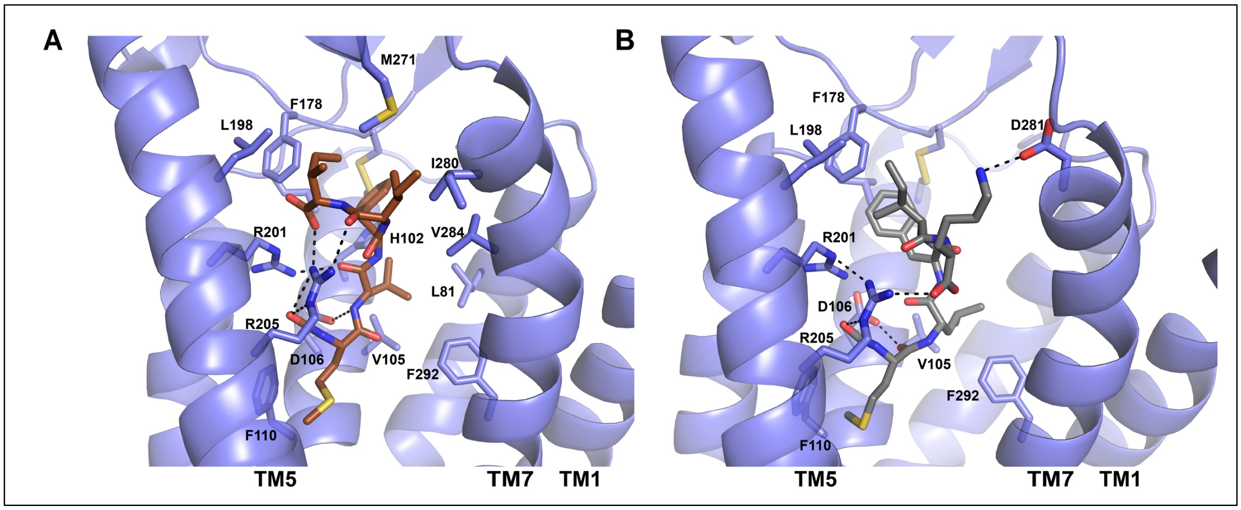

Supplementary Figure 6. Binding pockets of two formylpeptides in FPR2 by docking. (A) Binding pocket for fMLFII. Except for polar interactions with D106$^{3.33}$, R201$^{5.38}$ and R205$^{5.42}$ of FPR2, the four hydrophobic residues Leu, Phe, Ile and Ile in fMLFII form extensive hydrophobic interactions with residues L81$^{2.60}$, V105$^{3.32}$, F178, L198$^{5.35}$, M271, I280$^{7.31}$ and F292$^{7.43}$ of FPR2. **(B)** Binding pocket for fMLFIK. The last Lys residue forms a hydrogen bond with D281$^{7.32}$ of FPR2.

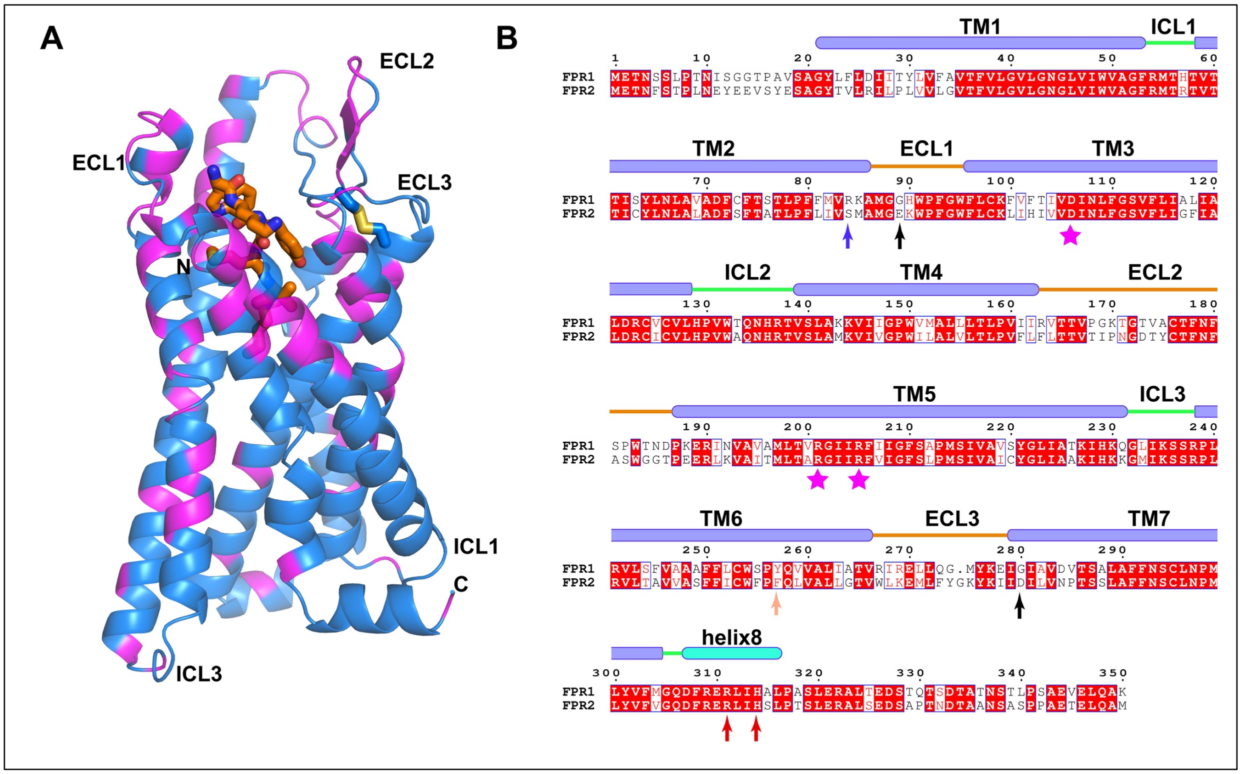

Supplementary Figure 7. Sequence difference between FPR1 and FPR2. (A) Structure of FPR2. Residues that are not conserved in FPR1 are shown in magenta. WKY is shown as orange sticks. ECL1-3, ICL1 and ICL3 are labeled. **(B)** Sequence alignment of human FPR1 and FPR2 with the secondary structural elements. Conserved residues are shown with red background. Residues E89 and D281$^{7.32}$ in FPR2 that are not conserved in FPR1 are indicated by black arrows. Residues R84$^{2.63}$ and Y257$^{6.51}$ in FPR1 that are not conserved in FPR2 are indicated by a blue arrow and a pink arrow respectively. Residues D$^{3.33}$, R$^{5.38}$ and R$^{5.42}$ that are important for recognizing N-formyl group are indicated by magenta stars. Residues R312 and H315 that are interact with Gβ are indicated by red arrows.

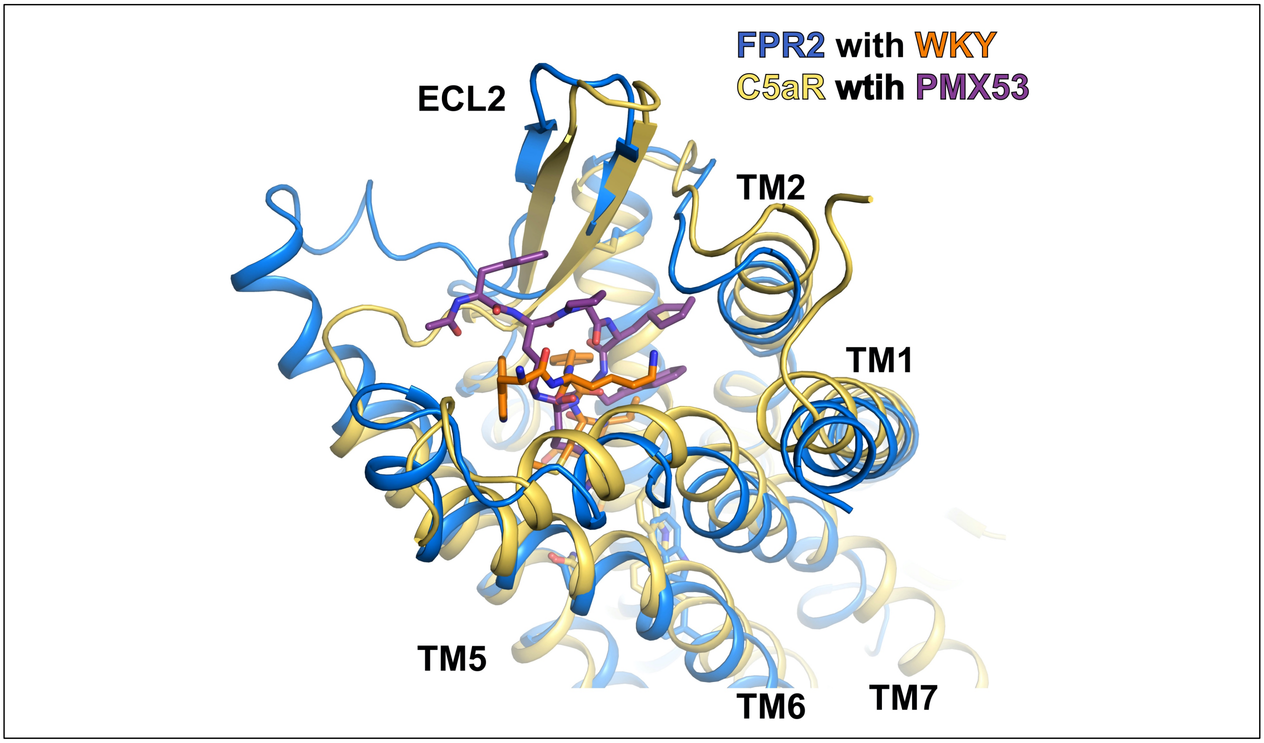

Supplementary Figure 8. Structural comparison of FPR2 (blue) with C5aR (yellow, PDB ID 6C1R). The peptide antagonist PMX53of C5aR is shown as purple sticks. The peptide agonist WKYMVm (WKY) of FPR2 is shown as orange sticks.

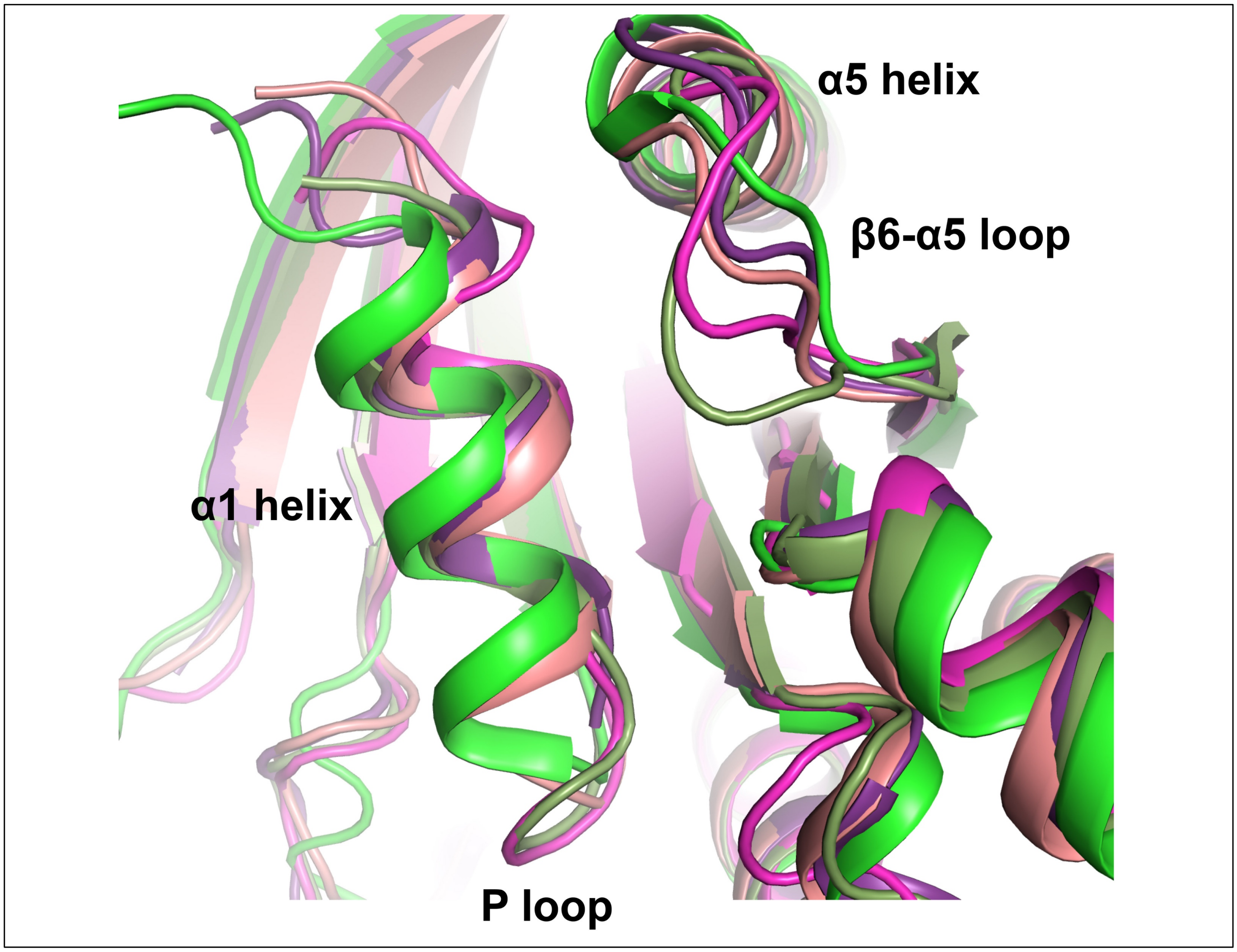

Supplementary Figure 9. Alignment of Gα$_i$ in the structures of FPR2-G$_i$ (salmon), µOR-G$_i$ (PDB ID 6DDE, magenta), A1AR-G$_i$ (PDB ID 6D9H, purple), CB1-G$_i$ (PDB ID 6N4B, dark green) and rhodopsin-G$_i$ (PDB ID 6CMO, green). The β6-α5 loop adopts different conformations in different structures.

SUPPLEMENTARY TABLES

SUPPLEMENTARY TABLE 1. IC_{50} values of six formylpeptides in the cAMP accumulation assays reported previously by *He et al., 2014*[*].

Formylpeptides	IC_{50} values, M	
	Wild type FPR2	FPR2 with $D281^{7.32}G$ mutation
fMLF	$2.5(\pm1.2) \times 10^{-6}$	$2.1(\pm0.4) \times 10^{-7}$
fMLFE	$2.6(\pm0.9) \times 10^{-4}$	$1.4(\pm0.6) \times 10^{-6}$
fMLFW	$6.4(\pm0.6) \times 10^{-7}$	$9.4(\pm0.6) \times 10^{-6}$
fMLFK	$5.1(\pm0.8) \times 10^{-8}$	$8.4(\pm0.7) \times 10^{-7}$
fMLFIK	$5.2(\pm1.6) \times 10^{-8}$	$2.1(\pm0.3) \times 10^{-7}$
fMLFII	$6.9(\pm1.3) \times 10^{-9}$	$4.1(\pm1.7) \times 10^{-9}$

[*]: He, H. Q., Troksa, E. L., Caltabiano, G., Pardo, L. & Ye, R. D. Structural determinants for the interaction of formyl peptide receptor 2 with peptide ligands. *J Biol Chem* **289**, 2295-2306, doi:10.1074/jbc.M113.509216 (2014).

SUPPLEMENTARY TABLE 2. Primer sequences for cloning FPR2 with an N-terminal prolactin signalling peptide and FLAG peptide and a C-terminal oMBP-MBP-8xHis tag into pFastBac vector; introducing mutations in Gαi1 and FPR2; cloning scFv16 into pFastBac vector.

	Forward primer (5'→3')	Reverse primer (5'→3')
Prolactin-Flag	CGGGATCCACCATGGACA GCAAAGGTTCGTCG	Primer1: CAGATTCTCAGCTGAACCG TCGACATCATCATCATCTTT GTAATCGGAGACCACACCC TGGCAC Primer2: GGAGAAGTTGGTTTCCATG CTAGCACCCTGGAAGTACA GATTCTCAGCTGAACCGTC G
FPR2	ATGGAAACCAACTTCTCC ACTC	GCCTTCTTCGATTTTGCGC ATGCAGGTCCCTGGAACAG GACTTCGAGGGTACCAGGT GAAGCAGAATTGGCAGC
oMBP-MBP-His8	GCAAAAATCGAAGAAGG CAAACTG	CCCAAGCTTTTAGTGATGGT GATGGTGATGGTGATGCTTG GTGATACGAGTCTGC
FPR2-E89A	ATGGGAGCAAAATGGCC TTTTGGCTGGTTCCTGTG	CCATTTTGCTCCCATGGCCA TGGAGACAATGAGG
FPR2-D106A	GTGGTGGCCATCAACCTC TTTGGAAGTGTCTTCTTG	GTTGATGGCCACCACGATG TGAATTAACTTACACAG
FPR2-L109A	ACATCAACGCCTTTGGAA GTGTCTTCTTGATTGG	TCCAAAGGCGTTGATGTCC ACCACGATGTGAATTAACTT AC
FPR2-F110A	AACCTCGCTGGAAGTGT CTTCTTGATTGGTTTCATT G	ACTTCCAGCGAGGTTGATG TCCACCACGATGTGAATTA AC
FPR2-F178A	TGTACTGCCAACTTTGCA TCCTGGGGTGGCACCCCT GAG	CAAAGTTGGCAGTACAGTA TGTGTCCCCATTTGGAATAG
FPR2-F257A	GTTTCCCGCTCAACTGGT TGCCCTTCTGGGCACCGT C	CAGTTGAGCGGGAAACCAA CAGATGAAGAAAGAAGC
FPR2-D281A	ATCATTGCCATCCTGGTT AACCCAACGAGCTCCCT G	CAGGATGGCAATGATTTTGT ACTTGCCATAGAACAAC
FPR2-F292A	CCTGGCCGCCTTCAACAG CTGCCTCAACCCCATGCT TTAC	TGTTGAAGGCGGCCAGGGA GCTCGTTGGGTTAACCAG
scFv16-Tev-His8	GTTCAATTAGTAGAGTCG	Primer1:

	GGTG	GATGGCTAGCACCCTGGAA GTACAGATTCTCGTCGACC AGCTCCAGCTTGGTACCTG Primer2: AAATATGCGGCCGCTTAGTG GTGATGATGATGGTGGTGAT GGCTAGCACCCTGGAAG
DNGαi1-G203A	GTGGGAGCTCAGAGATC TGAGCGGAAGAAGTGG	TCTCTGAGCTCCCACATCA AACATTTTAAAATGAAG
DNGαi1-A326S	CACATGTTCCACAGATAC TAAGAATGTGCAGTTTG	ATCTGTGGAACATGTGAAG TGGGTGTATATTTCCTTTG
